# The Evolution of the My Way Section: From the 1990s to 2020 and Beyond

**DOI:** 10.1093/asjof/ojaa032

**Published:** 2020-06-16

**Authors:** Jeffrey M Kenkel

**Affiliations:** Department of Plastic Surgery, UT Southwestern Medical Center, Dallas, TX

The artistry of aesthetic surgery continues to evolve and relies heavily on plastic surgeons’ constant search for perfection and better ways to accomplish safe and predictable outcomes. The *Aesthetic Surgery Journal* (*ASJ*) has been the platform that many have chosen over the past several decades to describe their techniques and innovations.

In the mid-1990s, *ASJ* used several creative ways to highlight contributions to the field. “Panel Discussions” and “My Way” were published teleconferences allowing authors to share their techniques to solve many of the challenging problems we faced in our practices. “My Practice to Yours” and “Surgical Strategies” were published articles by experts in the field, dedicated to sharing the technical details of specific procedures.^1^

For example, in the March 1995 issue of *ASJ*, Malcolm Lesavoy and Joel Feldman both shared their approach in correcting “Witch’s Chin.” ^2,3^ Dr. Lesavoy preferred a dermal flap technique to Dr. Feldman’s direct excision approach. Both described the rationale for their preferred choice and the details of the procedure. These papers exemplified articles found in the “Practice Forum” of the then known *Aesthetic Surgery Quarterly.* The clinical relevance and desire to learn technical details made this section quite popular.

In his Editorial in 2014, Dr. Foad Nahai acknowledged the historical importance of “how to” articles and developed a new section of the journal entitled “My Way.” As he described it, “My Way” content will include tips, tricks, and innovations that may not be lengthy enough for a full-length article yet are useful and worth sharing with our readers.” ^4^ Dr. Jamil Ahmad was appointed Section Editor and has done an excellent job at inviting, curating, and in some cases coauthoring “My Way” articles to ensure that they remained true to their original intent. Since this section’s inception, we have had 23 articles published in *ASJ*, many of which have included video content. The evolution of My Way articles has been underpinned by the increased popularity (and availability) of video content and has created an even greater opportunity to learn the author’s techniques in the explicit detail.

No longer does one have to hire a production company to film a procedure. Most of our phones have high-quality cameras, allowing us to easily film “teaching moments,” bringing our procedures to life for our reader, enabling us to teach our colleagues and to help educate plastic surgery residents and Fellows. These articles are technique-centered and include a small series discussion and an accompanying video.

Last year, we saw the launch of a new open-access journal, *Aesthetic Surgery Journal Open Forum*. I am honored to serve as Editor in Chief of *ASJ Open Forum* that has already become known for rapid peer review and broad dissemination of articles to a global audience. *ASJ Open Forum is* a sister journal to *ASJ* and is a peer-reviewed, open-access journal that targets preliminary work, case reports, Reviews, and technique-based submissions such as Featured Operative Techniques, My Way, and Video Review articles. During the first year of the journal, we published 3 My Way articles educating our readers on vaginoplasty and breast surgery.

We hope you will consider the opportunity to publish your technique and expertise in My Way so that our global readers can learn from your expertise. The opportunity to share your experience in written and video formats has never been easier. Given the recent limitations of travel and meeting presentations, video articles and detailed reviews in My Way provide our readers with opportunities to learn the nuances of surgical procedures for our patients. My Way articles should include a video and photographs (a minimum of 1 pre and postoperative photographs) and generally include 1000 to 1500 words, up to 5 figures and tables, and up to 35 references. More information can be found at https://academic.oup.com/asjopenforum/pages/General_Instructions. To submit your My Way manuscript, please visit https://mc.manuscriptcentral.com/asjof.

These past few months have been challenging times for us all. We are grateful that our publisher, Oxford University Press, has provided several resources that make publication easier.

Authors in many developing countries may be eligible for free or discounted publication charges. A complete list of countries can be found here: https://academic.oup.com/journals/pages/librarians/developing_countries/participating_countries. Also, *ASJ Open Forum* reviewers will also receive a 5% publication discount as our thanks for supporting the journal. We remain committed to publishing high-quality content and if we’ve learned one thing during COVID-19, it’s that video, Zoom, and other online educational tools are the future and an excellent accouterment to traditional learning.^5^

The Department of Plastic Surgery at UT Southwestern, where I have the privilege to serve as Professor and Chair of Plastic Surgery, has a long-established history of education and I have personally had the honor of working with some of the brightest residents and fellows in their earliest years. As their careers evolve and I watch them move from training into practice, present at national and international meetings, and begin to share what they’ve learned with the next generation, it gives me joy to see their professional development and evolution. In the same way, I am excited to carry the baton and help the My Way section continue to evolve in its new home, *ASJ Open Forum*, and I’m calling on you share your greatest learning moments so together we can pay that knowledge forward and continue this educational cycle.
